# Low Velocity Impact Response and Tensile Strength of Epoxy Composites with Different Reinforcing Materials

**DOI:** 10.3390/ma13143059

**Published:** 2020-07-08

**Authors:** Sebastian Sławski, Małgorzata Szymiczek, Jarosław Kaczmarczyk, Jarosław Domin, Eugeniusz Świtoński

**Affiliations:** 1 Department of Theoretical and Applied Mechanics, Silesian University of Technology, Konarskiego 18a, 44-100 Gliwice, Poland; malgorzata.szymiczek@polsl.pl (M.S.); jaroslaw.kaczmarczyk@polsl.pl (J.K.); eugeniusz.switonski@polsl.pl (E.S.); 2 Department of Mechatronics, Silesian University of Technology, Akademicka 10a, 44-100 Gliwice, Poland; jaroslaw.domin@polsl.pl

**Keywords:** aramid fiber, carbon fiber, polyethylene fiber, impact, tensile strength, Young’s modulus, tensile test, composite, epoxy matrix

## Abstract

This paper presents the results of research concerning multilayered epoxy composites reinforced with different materials. The strength of multilayered composites depends, to a large extent, on the reinforcing material. The authors decided to compare the low velocity impact response and perform tensile strength tests on several composites, to ascertain the mechanical properties of the prepared composites. Five different reinforcing materials were provided for the research (two fabrics made from aramid fibers, two fabrics made from carbon fibers and one fabric made from polyethylene fibers). The composites were manufactured by the vacuum supported hand laminating method. The low velocity impact response tests were conducted with the use of a pneumatic launcher. Three strikers with different geometry (conical striker, hemispherical striker and ogival striker) were used. A comparison of the resulting damage to the composites after the impact of the strikers was based on the images obtained using an optical microscope; tensile tests were also performed. The experimental investigation showed significant differences in the mechanical properties of the composites, depending on the applied reinforcing material. It was found that, as a result of the impacts, less damage occurred in the composites which were characterized by a lower Young’s modulus and a higher tensile strength.

## 1. Introduction

Multilayered woven composites are being increasingly used in various applications; such as, among others, aviation, the space industry, shipbuilding, the energy industry (e.g., as a material for manufacturing wind turbine blades), pressure tanks or as energy absorbing panels (also for military purposes). Multilayered composites are popular because of their low weight, high strength and high stiffness [1]. Their low weight is important in many applications; for example, an aircraft with a lower weight would be more fuel efficient [2]. The use of composite materials in the aviation industry has significantly increased in recent years and composites now constitute more than 25% of the Airbus A380 and 50% of the Boeing 787 aircraft [3]. Another example is a soldier’s personal equipment [4]. Fiber reinforced composites are also used in combination with other construction materials such as steel or aluminum (e.g., in protective panels [5]). The use of light weight composite materials could also increase the mobility of special purpose vehicles [6]. Weight is important in the case of the special purpose vehicles, because this is one of the parameters that influence their efficiency of movement [7]. The mechanical properties of composites depend on the materials used to make them. Fibers are mainly responsible for both the load transfer and the mechanical properties of multilayered composites. However, the applied matrix material also has an influence on the properties of composites. Mayer et al. [8] compared the ballistic resistance of aramid composites with various types of thermoplastic matrix. Pach et al. [9] compared the impact resistance of composites reinforced with aramid fibers with various types of matrix (epoxy and styrene-butadiene-styrene). Reis et al. [10] showed that the introduction of nanoclay into the epoxy resin could increase the maximum impact load of an aramid fiber composite. Mucha et al. [11] showed that the mechanical properties (such as tensile strength and elongation at break) of the epoxy matrix could be increased by addition of the multi-walled carbon nanotubes into the resin. The mechanical properties of the composite depend on the directions in which the reinforcing fibers are arranged. Therefore, multilayered composite materials reinforced with continuous fibers are orthotropic materials. As the properties of composites depend on the applied materials, the materials are selected based on the application for the manufactured composite. Manufacturing of composites with hybrid reinforcement materials has become more and more popular in recent years. In the case of hybrid composites, more than one kind of reinforcing material is used. As has been shown in many papers in the literature, the proper combination of different reinforcing materials can improve the useful properties of the manufactured composite. Naik et al. [12] showed that the post impact compressive strength of glass-carbon epoxy composites could be increased by the proper combination of the reinforcing layers. Naik et al. [12] demonstrated that the energy absorbed by a composite during an impact also depends on the combination of the reinforcing layers. Sarasini et al. [13] investigated the low velocity impact response and flexural strength of a hybrid composite that was reinforced by aramid and basalt fibers, as well as composites reinforced only by aramid or basalt fibers. In both cases, the sequence of the reinforcing layers had an impact on the results obtained. From the point of view of a composite’s strength properties, the manufacturing method and proper adhesion between the fibers and the polymer matrix are also important. Poor adhesion between the reinforcing fibers and the composite matrix can greatly reduce the strength of a composite.

In all of the aforementioned applications of multilayered composites, they were exposed to the impact of elements with various geometries. This issue is important, because, as shown in the literature [12,14], the post impact strength of a composite is much smaller than its nominal value. The post-impact tensile strength of the epoxy/carbon composites that were tested by Mitrevski et al. [15] were about twice as small as that of undamaged samples. However, poor post-impact compressive strength is the greatest weakness of multilayered composites in terms of their residual properties [16]. Therefore, knowledge about damage to a composite, as a result of impact with elements of various geometries is important. Damaged composites often need to be repaired or replaced. Composite elements are exposed to low velocity impacts in all applications. After such an impact, the visible damage is usually either small or not visible at all on the surface of the composite; however, the internal structure is usually far more damaged [17]. This is especially dangerous in the case of composites, as they are not designed to take impact load into account. The total energy during an impact can be divided between the energy of the moving striker, the energy absorbed by shear plugging, the energy absorbed by the deformation of the secondary yarns, the energy absorbed by tensile failure of the primary yarns, the energy absorbed by delamination, the energy absorbed by matrix cracking, and the energy absorbed by friction [18]. Composite energy absorbing panels are often reinforced with aramid and polyethylene fibers [1]. These fibers are characterized by a relatively high strength and large elongation at break. Carrillo et al. [19] investigated and compared the behavior of aramid fabric and a composite with a thermoplastic matrix reinforced by aramid fabric during an impact. Khodadadi et al. [20] compared the impact response of epoxy/aramid and rubber/aramid composites. The response of E-glass and Dyneema epoxy composites that were subjected to low and high velocity impacts was compared by Reddy et al. [21]. They found that the composites that were reinforced by E-glass fibers failed due to shear cutting. The composite that was reinforced by the Dyneema fibers underwent global deformation with tensile stretching of the fibers. They also found that the composites that were reinforced with the Dyneema fibers absorbed considerably more impact energy than the composites that were reinforced with E-glass fibers. Zhu et al. investigated the quasi-static and dynamic compression of UHMWPE (ultra-high molecular weight polyethylene) fiber-reinforced polyurethane composites [22]. One of the disadvantages of polyethylene fibers is their melting temperature (approximately 144–152 °C) [23]. Energy absorbing composites that are reinforced with polyethylene fibers should be hybrid composites that are reinforced with more than one reinforcing material. Carbon fibers are often used as a reinforcing material that has high strength. These fibers are stiff, so they are often used in hybrid composites, which results in an increase in the stiffness of the whole structure [24]. Mitrevski et al. [25] compared the penetration depth and energy absorption of epoxy/carbon composites that were hit by strikers with various nose geometries. Ulven et al. [26] concluded that, due to the small variation in the ballistic limit and crack propagation, the penetration of carbon/epoxy panels by projectiles with various geometries is significantly dependent on the thickness of the panels. Fadhil et al. compared the low velocity impact response of epoxy composites with various reinforcement materials [27]. They reported that the impact force, the deformation of the composites and the impact energy were different for the various types of carbon reinforcement. The results presented by Ahmed et al. [28] showed the differences between the impact response of epoxy composites that were reinforced with either carbon or glass fibers.

In this paper, the authors decided to examine the low velocity impact response of multilayered composites with different reinforcing materials and determine some of their mechanical properties based on tensile tests. The authors also decided to ascertain the relation between the mechanical properties of the composites (such as the Young’s modulus and the tensile strength) and their low velocity impact response. Fabrics made from aramid, carbon and polyethylene fibers were used as the reinforcing materials. In Section 2, the authors present selected reinforcing materials and the material chosen for the composite matrix; the manufacturing process is also presented. In Section 3, the authors describe the research concerning the low velocity impact response of composites reinforced with different materials. Steel strikers with three different geometries were used in this research. The damage caused by the strikers were analyzed and compared, based on the images taken by an optical microscope. The results of the conducted tensile tests are presented in Section 4. The mechanical properties of the composites, such as the tensile strength, the Young’s modulus and the strain at break are compared and the discussion about the obtained results is presented in Section 5. The correlation between the striker’s geometry and the resulting damage to the composites is also presented. The correlation between the mechanical properties of the composites and the reinforcing fabric that was used is also described. The authors also noted the correlation between the applied reinforcing fabric and the resulting damage from a low velocity impact. The conclusions of the article have been summarized and presented in Section 6.

## 2. Materials and Preparing the Composites

The manufacture of multilayered composites can be performed using various methods. In this research, the authors decided to use the vacuum supported hand laminating method. This is one of the simplest methods of manufacturing composites, but it does require some experience to produce a defect-free composite; a bad laminating process can decrease the strength of the composite. LG285 epoxy resin was used as the matrix with the HG285 hardener, as determined by the manufacturer. The resin was mixed with the hardener in a 100:40 ratio; the mechanical properties of the hardened matrix, as declared by the manufacturer, are presented in Table 1.

The strength of multilayered composites varies; the reinforcing fibers play a major role in the transfer of the load by a composite. One of the key aspects of the mechanical properties of a multilayered composite is the appropriate selection of the reinforcing fibers. In this paper, the authors decided to investigate epoxy composites with five different types of reinforcement. The reinforcing fabrics that were selected are made from different fibers and have both different weave and weight. In this research, the reinforcement that was used took the form of:

- Aramid fiber (Twaron 2200) fabric with a twill weave and a weight of 305 g/m^2^;
- Aramid fiber (Kevlar 49) fabric with a basket weave and a weight of 470 g/m^2^;
- Polyethylene fiber (Dyneema SK 60) fabric with a plain weave and a weight of 125 g/m^2^;
- Carbon fiber (Pyrofil TR30 S) fabric with a twill weave and a weight of 200 g/m^2^;
- Carbon fiber (HR 12K) fabric with a twill weave and a weight of 592 g/m^2^.

The mechanical properties of the fibers that were used in the selected fabrics are presented in Table 2.

**Figure 1:**
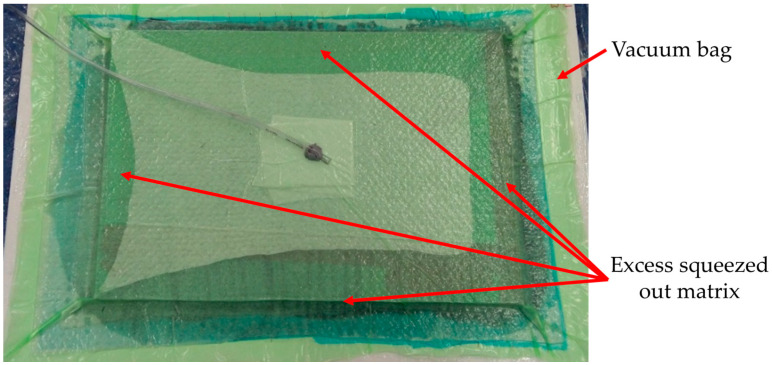
Squeezed out excess of the matrix during preparation of the composite by the vacuum supported hand laminating method.

For the symmetrical fabrics, all of the reinforcing layers were laid in the same orientation. For the fabrics that were not completely symmetrical (small differences in the fabric setting between the directions in which the fibers are arranged), the layers were stacked alternately (the fabric was rotated by 90° every layer). The manufacturing process was conducted with −68 kPa of vacuum pressure support. The vacuum support meant that the excess matrix was squeezed out of the composite. The amount of the resin that was squeezed out from between the reinforcing layers is shown in Figure 1.

Leaving the excess matrix between the reinforcing layers of the composite would increase the percentage volume of the matrix in the composite, and this would decrease the composite’s strength.

The laminated composite panel was left under the vacuum inside a bag for 3 h. After this time had elapsed, the pressure in the bag was returned to atmospheric pressure. The composite panel was removed from the bag the next day. Samples were cut from the prepared composite panels; the samples were then placed inside the SLW53STD laboratory dryer and kept at a temperature of 80 °C for 2 h. This manufacturing process was conducted for all of the prepared epoxy composites.

## 3. Low Velocity Impact Response

**Figure 2:**
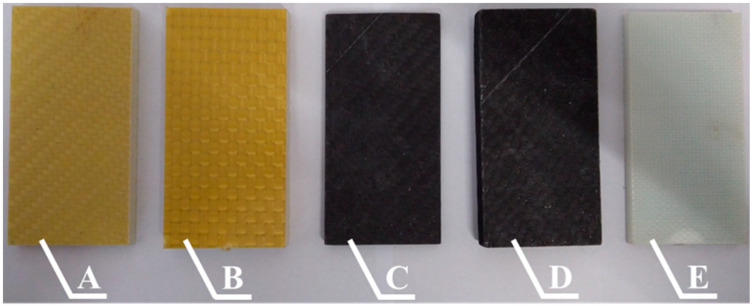
Samples cut from epoxy composites with sixteen layers of reinforcing fibers: **A**—Twaron 2200, **B**—Kevlar 49, **C**—Pyrofil TR30 S, **D**—HR 12K, **E**—Dyneema SK 60.

The research into the low velocity impact response was then conducted on the epoxy composite panels, which consisted of sixteen reinforcing layers and the manufacturing technology is as described in Section 2. As mentioned in Section 2, in the case of the symmetrical fabrics (reinforced by Twaron 2200, Pyrofil TR30 S, HR 12K fibers), all of the reinforcing layers were laid in the same orientation. In the cases when the fabrics were not completely symmetrical (small differences in the fabric setting between the directions in which the fibers are arranged), the layers were stacked alternately (the fabric was rotated by 90° every layer). The samples were cut parallel to the directions in which the reinforcing fibers were arranged. Samples with dimensions of 50 × 100 mm (height × width) were cut from the prepared composite panels. As mentioned in Section 2, the samples were subjected to the matrix hardening process. Prepared samples are shown in Figure 2.

**Figure 3:**
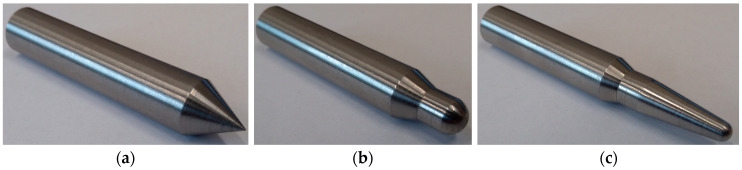
The strikers used in the experiment: (**a**) conical striker, (**b**) hemispherical striker, (**c**) ogival striker.

**Figure A1:**
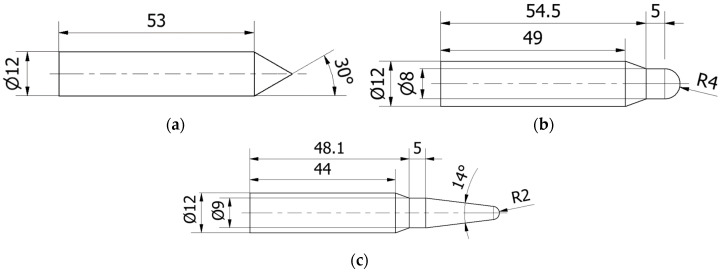
Geometries of the strikers: (**a**) conical, (**b**) hemispherical, (**c**) ogival.

Based on the specifications in the literature [25,34,35,36] and the technical requirements of the test stand, the geometry of the three strikers (conical striker, hemispherical striker and ogival striker) was developed. The strikers were designed with dimensions that allowed them to be the same weight (about 49 g). This was important as this means they will have the same impact energy during an impact at the same velocity. This in turn allowed the authors to assess the impact of the striker’s geometry on the resulting damage to the composites. The strikers were made from S235JR steel. The dimensions of the strikers are shown in Figure A1 and the manufactured strikers are shown in Figure 3.

The length and weight of each striker were measured and are shown in Table 3.

**Figure 4:**
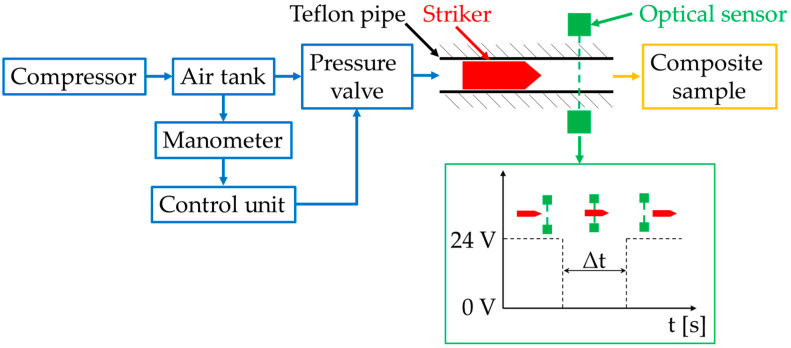
Scheme of the test stand with the pneumatic launcher used in the experiment.

The research concerning the low velocity impact response of the composites was conducted with the use of a pneumatic launcher. This device consisted of several main parts, such as a compressor, an air tank with a manometer, a control unit, a pressure valve, a Teflon pipe (Teflon minimizes the friction between the striker and pipe) and an optical sensor. The scheme of the launcher is presented in Figure 4.

The real-time controller (NI cRIO-9022 with digital input/output module and analogue input module) was used as a control unit to set the initial pressure P_0_ in the air tank. It was also used to open the pressure valve which releases the compressed air from the air tank to the Teflon pipe. Before the air was released from the air tank, the striker was placed inside the pipe. When the compressed air from the air tank was released, the striker inside the pipe started to accelerate as long as the air from the air tank was decompressing inside the pipe. A precise control unit enabled the engineers to achieve a repeatable velocity of the striker. The optical sensor placed at the end of the pipe was used to measure the striker’s velocity. Its method of operation is also presented in Figure 4. The optical sensor’s voltage signal was observed using the oscilloscope. When the striker starts to intersect the light beam, the value of the signal decreased from 24 to 0 V. At the moment when the striker no longer intersected the optical beam, the voltage signal increased from 0 to 24 V. Measuring the time between these two changes in the signal value provided information about the time in which striker broke the light beam of the optical sensor.

The velocity of the striker was calculated based on the ratio between the striker’s length to the time in which the striker broke the light beam. The striker’s velocity could be changed by changing the initial pressure P_0_. Due to the various shapes of the strikers’ head geometry, which affected the air resistance of the strikers, the initial pressure P_0_ was set individually for each striker to achieve a similar velocity at the time of impact. The composite samples were mounted at the end of the pipe; they were stuck to a 10 mm thick polyethylene base plate and mounted on a rigid steel frame. The measured values of the striker’s velocity and kinetic energy before impact are presented in Table 4. The kinetic energy was repeatable for each type of the strikers’ geometry for all the types of the applied reinforcing material. As the standard deviation was small, it could be assumed that the differences in the strikers’ kinetic energy had a negligible influence on the obtained results.

**Figure 5:**
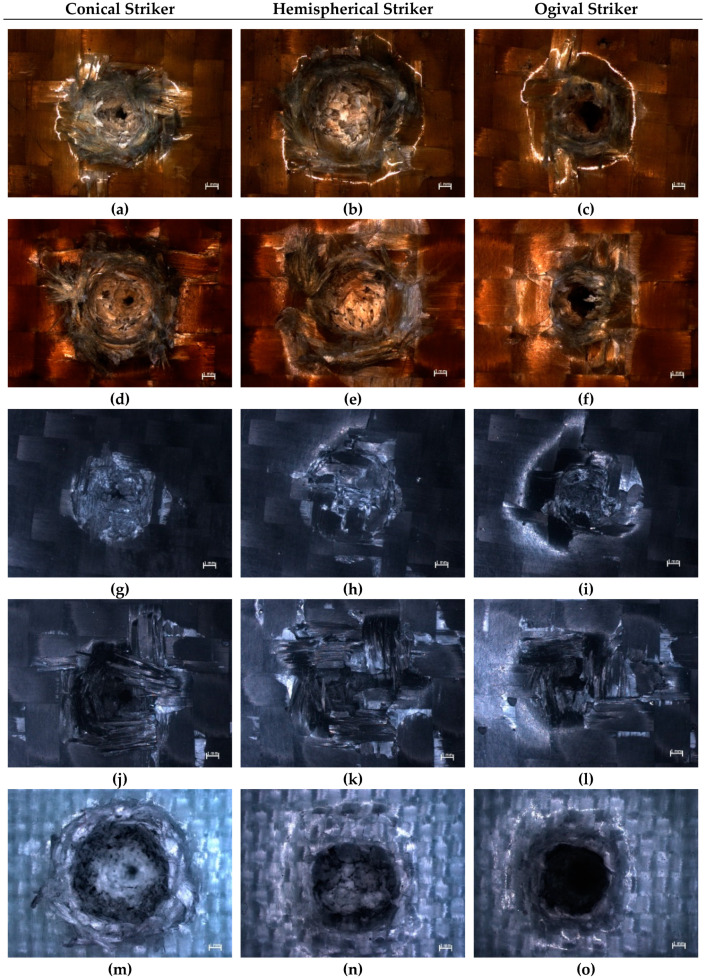
Microscopic images of damage caused by the strikers with various geometry in the epoxy composites with reinforcement made from the following fibers: (**a**–**c**) Twaron 2200, (**d**–**f**) Kevlar 49, (**g**–**i**) Pyrofil TR30 S, (**j**–**l**) HR 12K, (**m**–**o**) Dyneema SK 60.

A comparison of the resulting damage to the composite samples after impact was made from the images obtained from the optical microscope Discovery V12 (Carl Zeiss Microscopy GmbH, Jena, Germany) (with 8× zoom). The images presenting the resulting damage to the epoxy composites with various reinforcing materials due to the striker’s geometry are shown in Figure 5.

The diameters of the damaged areas presented in Figure 5 were measured and presented in Table 5. The diameters of the damaged areas were measured on the first reinforcing layer on the side of the impact. The measured area included the cavity and any visible damaged fibers (such as fibers ripped out from the matrix around the cavity).

Independent of the type of reinforcing material used, the least amount of damage was formed after the impact of the hemispherical striker (Figure 5b,e,h,k,n). Despite the relatively large diameter of the damage, which was caused by the striker’s geometry, the damage crater was shallow. The damage to the samples that were hit by the hemispherical striker was characterized by a large quantity of compressed and crushed fibers inside the cavities. The samples where carbon fibers had been used as reinforcement (Figure 5h,k) were characterized by brittle damage. The carbon fibers were not stretched over a large range, but they had been broken and ripped out of the matrix around the point of impact. This was especially visible in the sample shown in Figure 5k; in this case, the damaged area (diameter of 12.6 mm) in the first reinforcing layer on the impact side was larger than in the case of the samples where aramid fibers were used for the reinforcement (Figure 5b,e—diameter of 12.2 mm).

In the samples in which Twaron 2200 (Figure 5b) and Kevlar 49 (Figure 5e) fibers were used as the reinforcing material, it could be seen that, after the impact of the hemispherical striker, a large number of fibers were compressed inside the cavity. The damage to the epoxy composite reinforced by aramid fibers was characterized by a plastic form of damage—the cavity reflects the striker’s geometry. The damage to the composite reinforced by Dyneema SK 60 (Figure 5n) fibers was similar (the form of damage was also plastic). The deformation of the composite in the case of the hemispherical striker’s impact was the biggest in that case; the cavity also reflected the striker’s geometry.

An analysis of the images of the damage formed as a result of the conical striker’s impact, showed large differences between the composites made with various reinforcing materials. The formed cavity is the smallest in the composite reinforced by Twaron 2200 fibers (Figure 5a). In comparison with the sample reinforced by Kevlar 49 fibers (Figure 5d), both the diameter of the cavity and the amount of fibers ripped out of the matrix were smaller in the case of the sample reinforced by Twaron 2200 fibers. The diameter of the damaged area is 11.4 mm for the sample with reinforcement made from Twaron 2200 fibers and 12.1 mm for the sample with reinforcement made from Kevlar 49 fibers. The samples reinforced by carbon fibers (Figure 5g,j) were characterized by a smaller diameter of the damaged area (8.6 mm in case of the sample reinforced by Pyrofil TR30 S fibers and 10.6 mm in case of the sample reinforced by HR 12K fibers). In the case of the sample reinforced by Pyrofil TR30 S fibers (Figure 5g) there was no sign of the fibers having been ripped out of the matrix. The damage to this sample was characterized by a large diameter of the cavity at the first layer from the impact side. The damage to the further reinforcing layers was similar to a highly narrowed taper. The sample reinforced by HR 12K fibers (Figure 5j) was characterized by similar damage. However, in this case, there were a lot of crushed fibers which had been ripped out of the matrix around the impact point (diameter of damaged area is 10.6 mm). The sample reinforced by Dyneema SK 60 fibers (Figure 5m) was characterized with the most damage (diameter of damaged area is 12.2 mm); the material deformed plastically. A large amount of the reinforcing fibers were pushed out from the cavity; these fibers created a characteristic ring. Despite the large deformation, there were no signs of the fibers having been ripped out of the matrix around the cavity.

The damage caused by the impact of the ogival striker was similar to the damage caused by the conical striker. In the case of the samples that deformed plastically (Figure 5c,f,o), the pointy end of the striker burrowed in between the reinforcing fibers and pushed them sideways. This phenomenon was much less apparent in the composites reinforced by carbon fibers (Figure 5i,l), where the damage was brittle. Independent of the type of reinforcing material, the fibers were ripped out of the matrix. The sample reinforced by Dyneema SK 60 fibers (Figure 5o) was an exception because there was no sign of the fibers having been ripped out of the matrix. However, in this sample, the largest deformation around the impact point was observed (diameter of 11.4 mm). The material which was displaced by the striker created a characteristic ring around the impact point. The diameter of this deformation was larger than the diameter of the area in which the fibers were ripped out of the matrix in the other samples hit by the ogival striker (see Table 5). During the analysis of the images presented in Figure 5 and damaged areas diameter presented in Table 5, some similarities could be seen. Independent of the kind of reinforcing material (aramid, carbon), the use of fabrics with a higher weight (made from Kevlar 49 and HR 12K fibers) resulted in more damage (a lot of fibers ripped out of the matrix around the impact point) in the epoxy composites after a low velocity impact, than in the case of the same kind of the reinforcing materials but with a lower weight (fabrics made from Twaron 2200 and Pyrofil TR30 S fibers). The epoxy composite that was reinforced with the fabric with the lowest weight (Dyneema SK 60) was characterized by the absence of this type of damage (fibers ripped out of the matrix).

**Figure 6:**
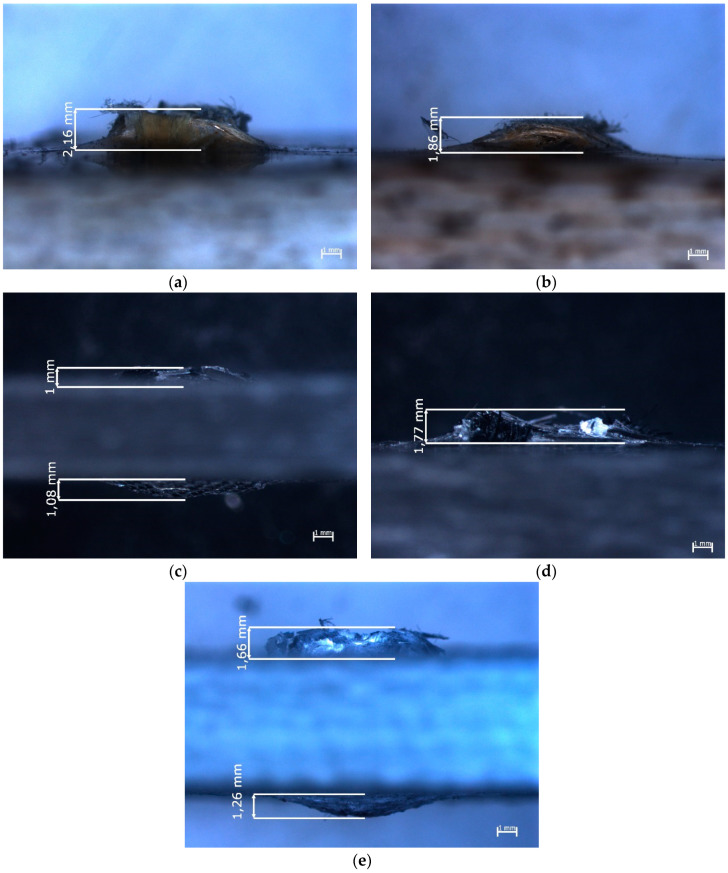
Epoxy composites with reinforcement made from the following fibers: (**a**) Twaron 2200, (**b**) Kevlar 49, (**c**) Pyrofil TR30 S, (**d**) HR 12K, (**e**) Dyneema SK 60, after impact of the ogival striker—perpendicular view point to the impact axis.

Images of the damage to the composites caused by the impact of the ogival striker are shown in Figure 6. Those images were taken from a view-point that was perpendicular to the impact axis.

**Figure 7:**
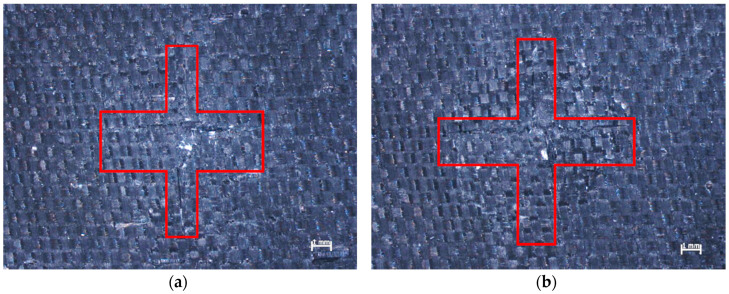
Epoxy composites reinforced by Pyrofil TR30 S fibers after impact from: (**a**) conical striker, (**b**) ogival striker—view from the opposite side to the impact side.

In the case of the sample in which Twaron 2200 fibers (Figure 6a) were used as reinforcement, the fibers that were ripped out of the matrix were narrowly focused around the cavity. The fibers that were ripped out of the matrix protruded less than in the case of the sample that was reinforced by Kevlar 49 fibers (Figure 6b). However, as can be seen in Figure 5f the diameter of the area in which the fibers had been ripped out of the matrix was larger in the case of the composite reinforced by Kevlar 49 fibers (11 mm), than in the case of the sample reinforced by Twaron 2200 fibers (9.8 mm). In the case of the samples in which carbon fibers were used as the reinforcing material (Figure 6c,d), the fibers that were ripped out of the matrix protruded less than in the case of the samples reinforced by aramid fibers. The carbon fibers that were ripped out from the matrix had been crushed (Figure 6c,d). In the image of the sample reinforced by Pyrofil TR30 S fibers (Figure 6c), a bulge on the opposite side to the impact side can be observed. A similar bulge can be observed in the image of the sample reinforced by Dyneema SK 60 fibers (Figure 6e). Despite the same number of reinforcing layers in all of the samples, the composites reinforced by fabrics made from Pyrofil TR30 S and Dyneema SK 60 fibers were the thinnest, which may have affected the formation of these bulges. Taking into account the bulges formed at the opposite side to the impact side, the damage to these composites could be much more serious than it seems. In the case of the samples reinforced by Pyrofil TR30 S fibers, damage on the opposite side to the impact side was also visible; this damage is shown in Figure 7.

Deformation of the regular structure of the fibers can be observed in Figure 7; two cracks in the perpendicular directions are also visible. Theses directions are parallel to the direction of the reinforcing fibers and the intersection point of the cracks also intersects the impact axis. This damage proves that the primary yarns are subjected to the highest load. In both cases presented in Figure 7, the damage was located at the bulges.

**Figure 8:**
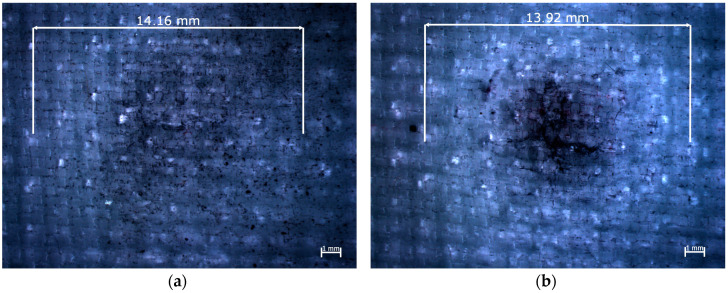
Epoxy composites reinforced by Dyneema SK 60 fibers after impact from: (**a**) conical striker, (**b**) ogival striker—view from the opposite side to the impact side.

In the cases of the bulges formed on the opposite side to the impact side in the sample reinforced by Dyneema SK 60 fibers (Figure 8), similar cracks were observed.

However, in this case, the lengths of the cracks were smaller and located around the striker’s impact point. It could be observed that the brittle damage to the carbon fibers occurred in the perpendicular directions over a longer distance (Figure 7) than in the case of the plastically deformed polyethylene fibers (Figure 8).

The delamination of the multilayered composites is very significant, as it is one of the energy absorbing phenomena that occur during the impact. Delamination is also dangerous for the structure that was hit because it reduces the strength of the composite. Delamination is often hard to detect; however, in some types of the reinforcing materials, which are partly transparent, delamination could be seen as white rings that formed around the impact area. In the conducted research in the case of the composite sample reinforced by Dyneema SK 60 fibers (Figure 8), white rings are visible around the impact point. It was noted that, independent of the striker’s geometry, the diameter of the white rings was around 14 mm. It was assumed that delamination occurred in all of the samples, but it was not visible in some of the samples because aramid and carbon fibers are not transparent.

## 4. Tensile Test

**Figure 9:**
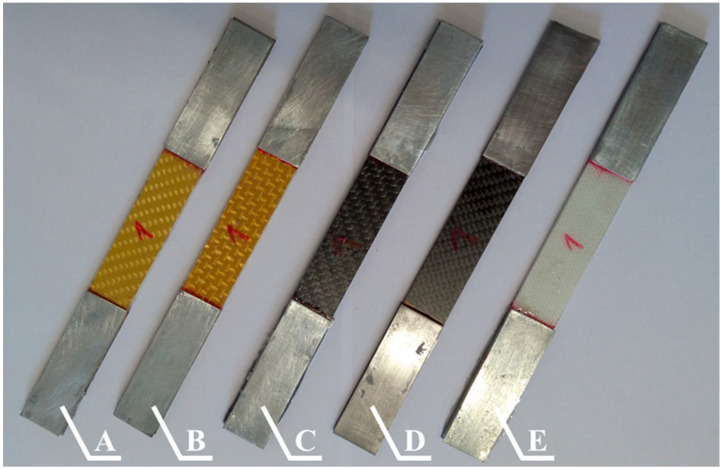
Selected epoxy composites samples (with steel covers) reinforced by the following fibers: **A**—Twaron 2200, **B**—Kevlar 49, **C**—Pyrofil TR30 S, **D**—HR 12K, **E**—Dyneema SK 60.

The composite samples used in the tensile tests were prepared in accordance with the process described in Section 2. The samples reinforced by fabrics made from Pyrofil TR30 S and Dyneema SK 60 fibers consisted of 10 reinforcing layers; the samples reinforced by the other fabrics consisted of 8 reinforcing layers. Samples with dimensions of 25 × 250 (width × length) were cut from the prepared composite plates. All of the composite materials were tested in the direction in which the reinforcing fibers were arranged. The tensile tests were conducted based on the standard [37] that are dedicated for woven composites. The tests were conducted with a 2 mm/min deformation speed. The tests were carried out on a “Zwick Z100” (ZwickRoell GmbH & Co. KG, Ulm, Germany) testing machine equipped with self-clamping jaws. The strain in the sample in the longitudinal direction was measured by a mechanical extensometer which was integrated into the testing machine. The length of the test sections was 90 mm. Steel covers with a thickness of 1 mm were glued to both ends of the composite samples; examples of the prepared composite samples are shown in Figure 9.

**Figure 10:**
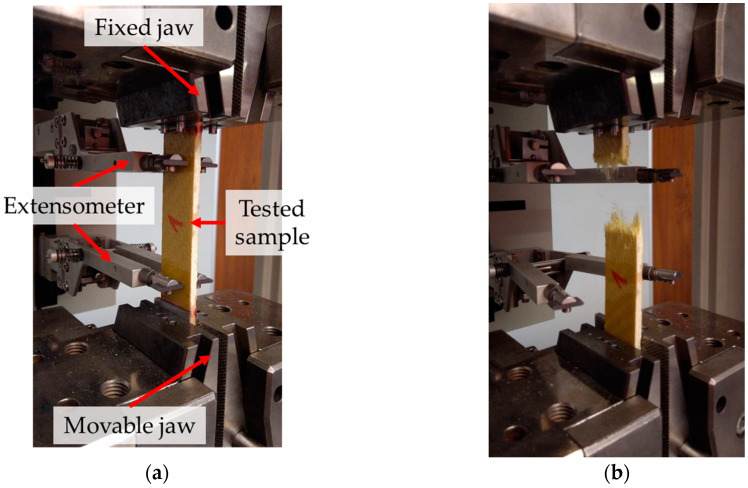
Tensile test of a composite sample reinforced by Twaron 2200 fibers: (**a**) sample (with active extensometer) placed in the jaws, (**b**) sample after rupture.

The tests were performed using three samples from each type of prepared epoxy composites. The upper stress boundary at which the extensometer stopped measuring the sample’s strain was set. Disconnection of the extensometer during the test was necessary to prevent it from being damaged at the moment when the sample was destroyed. This was due to the fact that multilayered composites do not have a yield point and their destruction is dynamic. Further deformation of the sample was measured based on the traverse displacement of the testing machine. A better solution for the sample strain measure is use of the digital image correlation system, but due to the testing machine construction it was not possible. The strain of all of the composite samples was measure by the extensometer, up to 200 MPa. As the stress–strain curve of the tested materials was almost linear, the strain at break could be calculated using the Young’s modulus of the sample. A composite sample reinforced by Twaron 2200 fibers during the test is shown in Figure 10.

**Figure 11:**
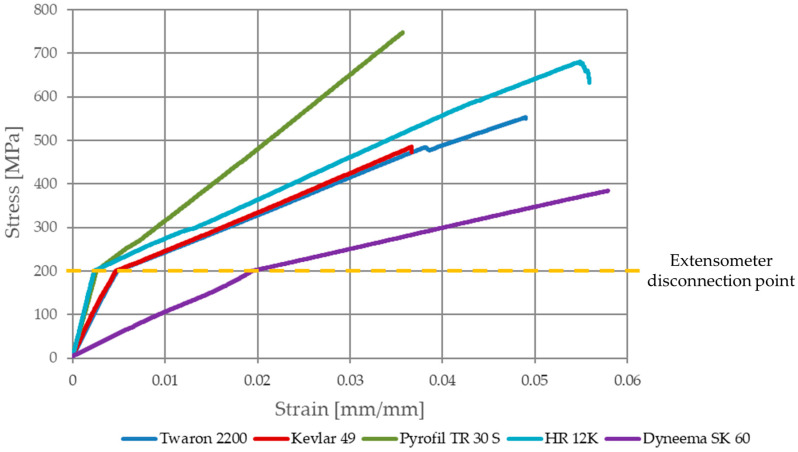
Stress–strain curves of the epoxy composites with various reinforcing materials.

Based on the conducted research, the tensile strength, the Young’s modulus and the strain at break were determined for all the types of composite samples. The selected stress–strain curves (one for each type of composite) of the samples recorded during the experiment are shown in Figure 11.

The characteristic change of the slope angle of the curves can be seen after the stress of the samples reached 200 MPa; this was due to the disconnection of the extensometer. After this stress value, the strain was measured based on the testing machine’s traverse displacement, which is the sum of: strain of the sample test section, displacement between the sample and steel covers (if occurs), displacement between steel covers and testing machine jaws (if occurs), stiffness of the testing machine and kinematic clearance. The Young’s modulus was calculated based on the first part of the stress–strain curves (based on the sample strain measured by the extensometer) presented in Figure 11 (before the slope angle of the curves changed). The Young’s modulus was determined at strain values of ε_1_ = 0.0005 and ε_2_ = 0.0025. In the case of the samples reinforced by HR 12K fibers, due to the high value of its Young’s modulus, this was determined at strain values of ε_1_ = 0.0005 and ε_2_ = 0.002. Such strain values were adopted because the ε_2_ strain value corresponds to the σ_2_ = 197.8 MPa stress value. If the strain value ε_2_ was adopted in the calculations, in accordance with the literature [37] (ε_2_ = 0.0025), then the calculated Young’s modulus will be burdened with an error resulting from the sample’s strain measurement from the traverse testing machine. Out of all of the composite samples reinforced by HR 12K fibers, only one was destroyed during the test. This was because the range of the machine is too small (its 100 kN load limit was exceeded).

It can be seen that the presented curves are almost linear; single nonlinear behavior of the stress-strain curves are correlated with damage to the polymeric matrix of the composites. Therefore, the calculation of the strain at break based on the Young’s modulus (which were determined based on the strain measured by the extensometer) was not burdened with a large error. The results of the conducted research are shown in Table 6.

**Figure 12:**
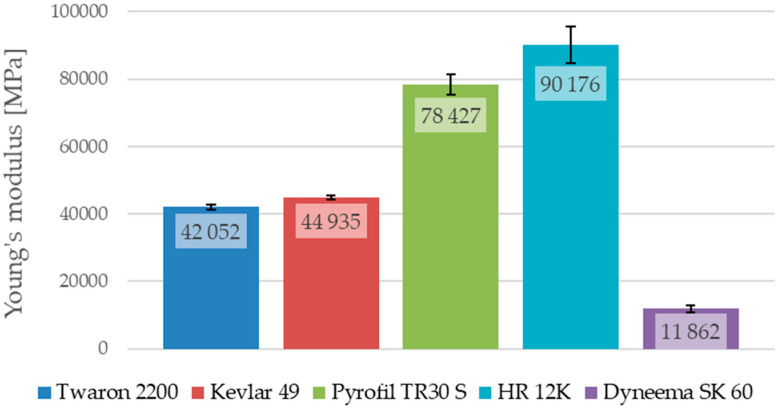
Average Young’s modulus of the epoxy composites with various reinforcing materials.

**Figure 13:**
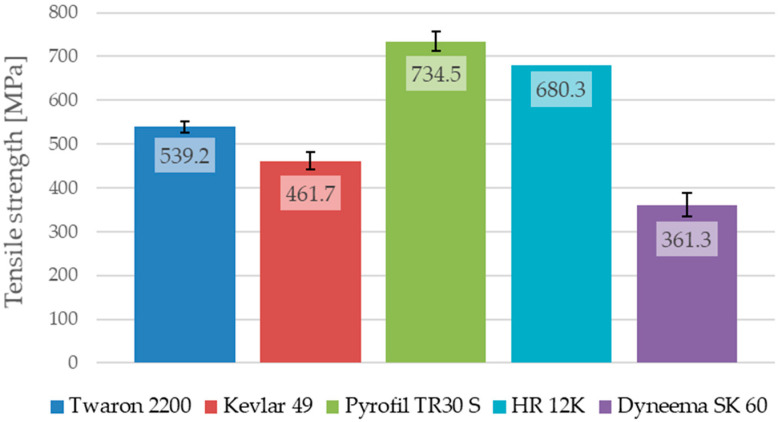
Average tensile strength of the epoxy composites with various reinforcing materials.

**Figure 14:**
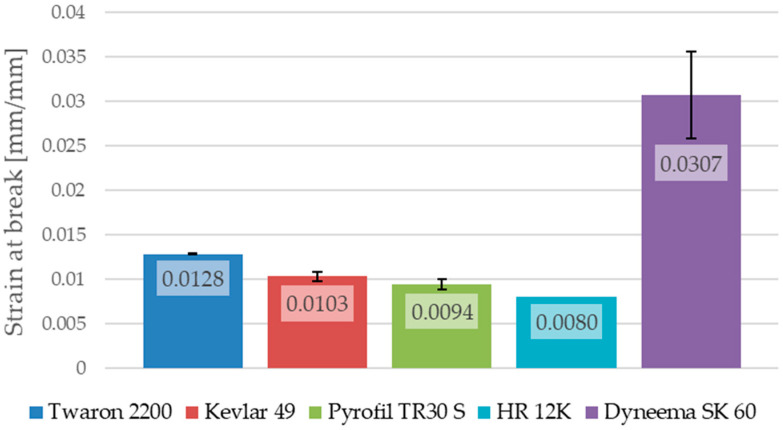
Average strain at break of the epoxy composites with various reinforcing materials.

The maximum stress of the samples reinforced by HR 12K fibers, which were not damaged during the test, were taken at the moment in which the testing machine’s load limit was reached. In the case of the samples reinforced by HR 12K fibers, the tensile strength and strain at break, obtained for the sample which was destroyed during the test, are given as an average value for this type of composite. The average values of the Young’s modulus, the tensile strength and the strain at break for the composites with various types of reinforcement are shown in Figure 12, Figure 13 and Figure 14.

The analysis of the results, presented in Table 6, shows that the results obtained for the composites with various types of reinforcement, but made from the same kind of reinforcing fibers, are different. The tensile strengths for the composite samples reinforced by aramid fibers are different to the samples reinforced by the Twaron 2200 and Kevlar 49 fibers; the Young’s modulus and strain at break are also different. The samples reinforced by Twaron 2200 fibers were characterized by a higher tensile strength (difference about 77.5 MPa) in comparison to the samples reinforced by Kevlar 49 fibers. They are also characterized by a slightly lower Young’s modulus (difference about 2.8 GPa) and a higher strain at break (difference about 0.0025). It is worth noting that, the fabric made from Twaron 2200 fibers is characterized by a lower weight than the fabric made from Kevlar 49 fibers, which could have had an impact on the strength properties of the multilayered composites that were prepared with the use of those reinforcing materials. The samples reinforced by Pyrofil TR30 S fibers are characterized by a much lower Young’s modulus in comparison to the samples reinforced by HR 12K fibers (difference about 11.7 GPa). The fabric made from HR 12K fibers has a much higher weight than the fabric made from Pyrofil TR30 S fibers. This correlation is identical to the case of the samples reinforced by aramid fibers. This has shown that the use of a reinforcing fabric with a higher weight results in a higher Young’s modulus of the composite.

As was mentioned, only one of the composite samples reinforced by HR 12K fibers was destroyed during the tensile test. Based on the obtained results, it can be stated that the tensile strength (difference about 54.2 MPa) and strain at break (difference about 0.0014) for these samples are smaller than in the case of the samples reinforced by Pyrofil TR30 S fibers. However, it is worth noting, that the sample reinforced by HR 12K fibers which was destroyed during the test is characterized by a lower Young’s modulus than the other samples reinforced by the same fibers (see Table 6). The strain of the other samples reinforced by HR 12K fibers at the moment when they reached the stresses presented in Table 6 (at the load limit of the testing machine) was lower. Therefore, it can be assumed that they would be damaged at a similar strain to the strain of the destroyed sample. Then their tensile strength would be much higher than in the moment when the test was stopped. The highest tensile strength was achieved for the samples reinforced by carbon fibers. The tensile strength of the samples reinforced by aramid fibers was about 1/3 lower than the carbon fiber samples. The lowest tensile strength was obtained for the samples that were reinforced by polyethylene fibers. Such low tensile strength of the epoxy composite reinforced by Dyneema SK 60 fibers could have been caused by poor adhesion between the epoxy matrix and the polyethylene fibers. Similarly, as in the samples reinforced by aramid fibers, the composite samples reinforced by carbon fibers are characterized by the same correlation. When a reinforcing fabric with a lower weight was used, the Young’s modulus was smaller and the tensile strength and strain at break were higher.

The samples reinforced by Dyneema SK 60 fibers are characterized by the largest strain at break (0.0307). This value is about three times greater than in the case of the samples reinforced by aramid or carbon fibers. The samples reinforced by Dyneema SK 60 fibers are also characterized with the lowest Young’s modulus (11.9 GPa). This is about four times lower than in the case of the samples reinforced by aramid fibers. The smallest strain at break was observed in the case of the samples reinforced by carbon fibers; for these samples, the Young’s modulus was the highest among all of the analyzed cases. This is about two times higher than in the case of the samples reinforced by aramid fibers. This is why carbon fibers are often used in hybrid composites to increase their stiffness [24].

**Figure 15:**
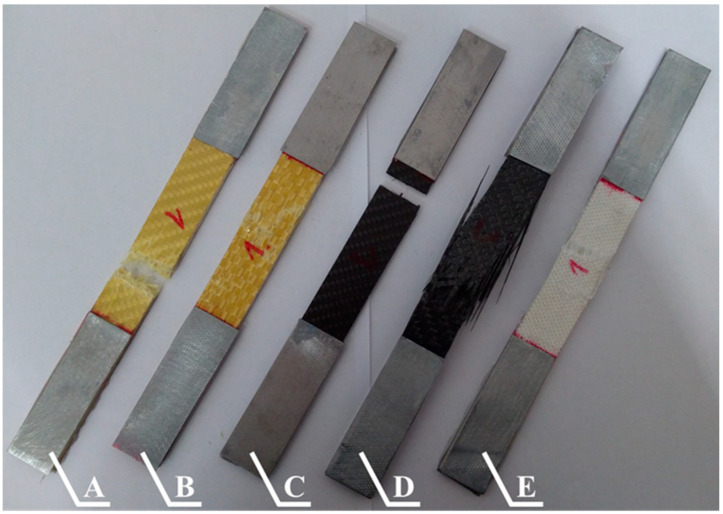
Selected epoxy composites samples with reinforcement made from the following fibers: **A**—Twaron 2200, **B**—Kevlar 49, **C**—Pyrofil TR30 S, **D**—HR 12K, **E**—Dyneema SK 60 after the tensile test.

The selected samples (each with a different reinforcing material) used in the tensile test are shown in Figure 15. Each of the tested composites is characterized by a different type of destruction. The destruction of each sample took place in the test section with a length of 90 mm. However, some samples were not completely destroyed (they were not ripped into two parts). The crack lines in the samples reinforced by Twaron 2200 fibers (Figure 15A) were perpendicular to the tensile direction; this sample was ripped. A large amount of the fibers ripped out from the second part of the sample are visible. The sample reinforced by Kevlar 49 fibers (Figure 15B) was not completely destroyed. There were a lot of broken fibers, but the composite did not lose its entire strength. In the case of the sample reinforced by Pyrofil TR30 S fibers (Figure 15C), the crack line was perpendicular to the tensile direction, as in the case of the sample reinforced by the Twaron 2200 fibers; however, the damage was different. There was no sign of the fibers having been ripped out of the second part of the tested sample (brittle damage). The damage to the samples reinforced by the HR 12K (Figure 15D) and Dyneema SK 60 (Figure 15E) fibers is characterized by the adhesion of the material between successive reinforcing layers being broken (delamination) and the single layers of the reinforcing fabrics having broken.

## 5. Discussion

In the cases with the same kind of reinforcing material, it was noted that less damage occurred (as a result of the striker’s impact) in the composites which are characterized by a lower Young’s modulus. In the composites with the same kind of reinforcing materials, less damage and a lower Young’s modulus were observed in the case of the composites reinforced by fabrics with a lower weight. The composite reinforced by polyethylene fibers is characterized by the largest deformation formed as a result of the striker’s impact. This composite is also characterized by the lowest Young’s modulus among all of the tested materials. The results described in the literature [21] show that the composites reinforced by Dyneema fibers deformed to a greater extent than the composites reinforced by E-glass fibers. The comparison between the composites reinforced by Dyneema and E-glass fibers presented in the literature [21] showed also that the composite reinforced by Dyneema fibers absorbed much more impact energy. Based on the mentioned paper and observed damage (deformation of the sample), the authors suppose that the composite reinforced by polyethylene fibers absorbed a much higher amount of the impact energy than the other tested composites. However, confirmation of this assumption requires further research. It was noted that the damage formed in the composites that were reinforced by aramid and polyethylene fibers was plastic damage. This plastic deformation of the composites reinforced with aramid fibers was also reported in the literature [38]. The resulting damage to those composites reflects the geometry of the striker. In the case of the composites reinforced by carbon fibers, a lot of crushed fibers were observed (brittle damage).

It is worth noting the cracks on the opposite side to the impact side in the case of the composites reinforced by Pyrofil TR30 S and Dyneema SK 60 fibers; these cracks occurred in the perpendicular directions to which the reinforcing fibers were arranged. The intersection of these cracks was at the intersection point with the striker’s impact axis. Hosur et al. [39] also reported such damage on the opposite side to the impact side in the case of carbon fiber reinforced composites. The same perpendicular cracks were reported in the case of the epoxy composites reinforced by aramid and basalt fibers [13]. In this paper in the literature, the length of the cracks depended on the impact energy (if the impact energy was greater, then the cracks were longer). Ulven et al. [26] noted that the penetration of the epoxy/carbon composites significantly depended on the composite’s thickness. In the conducted research, there were significant differences in the thickness of the epoxy/carbon composites. An additional study using the same thickness of the composites but with a larger number of the reinforcing layers of a fabric with a lower weight could be conducted. A composite with a higher number of reinforcing layers with a lower weight may be a better solution for the impact response, than a composite with the same thickness but reinforced by a fabric with a higher weight; this issue may be the subject of further research. During the analysis of the damage that was formed, it was worth considering delamination which makes a composite much weaker. In the conducted research, in the case of the composite sample reinforced by polyethylene fibers, white rings could be observed around the impact point. This is the area in which the connection between the adjacent reinforcing layers was damaged. The diameter of this damage was about 14 mm for all of the strikers. The numerical studies presented in the literature [40] have shown that the delamination occurred between the reinforcing layers, independent of the striker’s geometry. However, the diameter of the areas of delamination depends on the striker’s geometry [15]. The results presented in the literature [40] have also shown that the delamination area was larger when damage in the reinforcing layers was smaller. The internal damage, such as delamination, could be examined with the use of nondestructive tests [41,42,43,44]. The delamination area could be decreased by, for example, matrix modification. As is shown in the literature [45], incorporating polycaprolactone nanofibers reduced the area of delamination and could decrease the size of the damaged area in glass fiber reinforced polymer composites.

The damaged area in the first reinforcing layer on the impact side was the biggest in the case of the hemispherical striker’s impact; however, this is related to the striker’s geometry. It was also noted that the damage to the composite plates was shallowest (penetrating the smallest number of reinforcing layers) in the case of the impact from the hemispherical striker; there was a lot of crushed fibers in the cavity. If the geometry of the striker was pointier, then the amount of damage and the penetration depth of the composites were larger; this was because the pointed end of the conical and ogival strikers burrowed into the reinforcing fibers and pushed them sideways. The results presented in the literature [25,46] have also confirmed that the smallest penetration occurred in the case of the hemispherical striker.

The conducted tensile tests showed big differences between the mechanical properties of the composites with various reinforcing materials. The highest value of tensile strength was obtained for the composites reinforced by carbon fibers. For those same composites, the Young’s modulus was also the highest among the tested samples; however, the strain at break was the smallest. In the case of the composites reinforced by carbon fibers, the highest value of the tensile strength (734.5 MPa) was obtained for the composite reinforced by a fabric with a lower weight (Pyrofil TR30 S fibers). However, the Young’s modulus in this case (78.4 GPa) was lower than in the case of the composite reinforced by HR 12K fibers (90.2 GPa). The tensile strength of the samples reinforced by aramid fibers was about 1/3 lower than in the case of the composites reinforced by carbon fibers. As is in the case of the carbon fibers, the highest value of the tensile strength (539.2 MPa) was obtained in the case of the epoxy/aramid composite reinforced by a fabric with a lower weight (Twaron 2200 fibers). The Young’s modulus was also smaller in the case of the composite reinforced by a fabric with a lower weight. The Young’s modulus and tensile strength were different for the composites reinforced by fabrics made from the same kind of reinforcing fibers. In the case of the composites reinforced by a fabric with a lower weight, a higher tensile strength and a lower Young’s modulus were observed for the same kind of reinforcing fibers. In the cases of the aramid and carbon fibers, the tendency for the Young’s modulus and strain at break were the same as in the case of the fibers’ mechanical properties (e.g., the composite reinforced by fibers with a higher Young’s modulus had a higher modulus than the composite reinforced by a fiber with a lower Young’s modulus); however, there was a difference in the tensile strength. This is important because, as shown in Table 2, the fibers from which a fabric with a lower weight was made are characterized by a lower tensile strength than the fibers from which a fabric with a higher weight was made. Therefore, the case of the tensile strength tendency is the opposite; composites reinforced by fibers with a lower tensile strength (fabrics with a lower weight) have a higher tensile strength than composites reinforced by fibers with a higher tensile strength (fabrics with a higher weight). Based on this conclusion the authors deduce that this is related to the fabrics’ weight and weave. The conducted research has shown that the weight of the reinforcing fabric has an impact on the mechanical properties of the multilayered composites. The strength of the composite samples reinforced by polyethylene fibers was relatively low. It is thought that there was not a proper connection between the reinforcing fibers and the epoxy matrix. It is possible that the use of special agents to improve the adhesive bond between the reinforcing fibers and the epoxy matrix is required.

## 6. Conclusions

This article presents the results of the low velocity impact response and tensile test of the epoxy composites with different reinforcing materials. Based on the performed research, the authors determined the influence of the reinforcing material on the low velocity impact response and basic mechanical properties of the composites (such as tensile strength, Young’s modulus, strain at break). The authors also determined the dependency between the mechanical properties and the low velocity impact response of the tested composites. The conclusions from the research are as follows:

- The damage to the multilayered composites depended on the striker’s geometry. The least damage was caused by the hemispherical striker (the damage extended to a small number of the reinforcing layers). The damage caused by the strikers with a pointed end was much greater. A large number of the reinforcing layers were penetrated by the pointed strikers, because the pointed end of the striker burrowed between the fibers and pushed them sideways.
- In the case of the composites reinforced by the same kind of reinforcing fibers, the damage (as a result of a low velocity impact) to the composites reinforced by fabrics with a lower weight was less than in the case of the composites reinforced by fabrics with a higher weight.
- The composites reinforced by aramid and polyethylene fibers were characterized by plastic damage. The nature of the damage to the composites reinforced by carbon fibers was brittle damage.
- The tensile strength of the tested composites was the highest for the composites reinforced by carbon fibers. The tensile strength of the composites reinforced by aramid fibers was about 1/3 less. The lowest tensile strength was obtained for the composites reinforced by polyethylene fibers. The poor tensile strength of the epoxy composite reinforced by Dyneema SK 60 fibers could have been caused by poor adhesion between the epoxy matrix and the polyethylene fibers.
- The highest tensile strength and the lowest Young’s modulus, in the cases of the composites reinforced by aramid fibers and the composites reinforced by carbon fibers, were observed in the cases where the composites had been reinforced by fabrics with a lower weight.
- The damage caused as a result of the striker’s low velocity impact in the case of the composites reinforced by the same kind of reinforcing fibers was smaller when a fabric with a lower weight was used.
- As a result of the striker’s low velocity impact in the composites that were reinforced by the same kind of reinforcing fibers, less damage was observed in the cases where the composites had a higher tensile strength and a lower Young’s modulus (for the fabrics with a lower weight).

## Figures and Tables

**Table 1:** Properties of the epoxy resin LG285 with the HG285 curing agent [29].

Parameter	Units	Value
Flexural modulus	MPa	2700–3300
Tensile strength	MPa	75–85
Compressive strength	MPa	130–150
Elongation at break	%	5–6.5
Hardness in the Shore D scale	-	85

**Table 2:** The mechanical properties of the selected reinforcing fibers [23,30,31,32,33].

Parameter	Units	Twaron 2200	Kevlar 49	Pyrofil TR30 S	HR 12K	Dyneema SK 60
Elongation at break	%	2.9	2.4	1.8	1.1	3–4
Tensile strength	MPa	2930	3600	4120	4410	2400–3300
Tensile modulus	GPa	102	124	235	375	65–100

**Table 3:** Length and weight of the strikers used in the experiment.

Striker’s Geometry	Length (mm)	Weight (g)
Conical	63.1	48.92
Hemispherical	63.4	48.98
Ogival	74.3	48.97

**Table 4:** Velocity and kinetic energy of the strikers before impact with the composite sample.

Striker Geometry	Reinforcing Material	Velocity (m/s)	Kinetic Energy (J)	Average Kinetic Energy (J) (Standard Deviation)
Conical	Twaron 2200	31.24	23.9	23.6 (0.42)
	Kevlar 49	30.93	23.4	
	Pyrofil TR30 S	30.93	23.4	
	HR 12K	30.93	23.4	
	Dyneema SK 60	31.55	24.4	
Hemispherical	Twaron 2200	31.54	24.4	24.8 (0.65)
	Kevlar 49	31.54	24.4	
	Pyrofil TR30 S	32.35	25.6	
	HR 12K	32.35	25.6	
	Dyneema SK 60	31.70	24.6	
Ogival	Twaron 2200	31.75	24.7	24.2 (0.65)
	Kevlar 49	31.22	23.8	
	Pyrofil TR30 S	31.48	24.2	
	HR 12K	32.03	25.1	
	Dyneema SK 60	30.96	23.4	

**Table 5:** Diameter of the damaged area on the first reinforcing layer from the side of the impact.

Striker Geometry	Diameter of the Damaged Area (mm)				
	Twaron 2200	Kevlar 49	Pyrofil TR30 S	HR 12K	Dyneema SK 60
Conical	11.4	12.1	8.6	10.6	12.2
Hemispherical	12.2	12.2	9.5	12.6	12.2
Ogival	9.8	11	10.4	11.2	11.4

**Table 6:** Dimensions of the composite samples and their mechanical parameters calculated based on the conducted tensile tests.

Reinforcing Material	Sample	Width (mm)	Thickness (mm)	Cross-Section Area (mm^2^)	Tensile Strength σ (MPa)	Young’s Modulus E (MPa)	Strain at Break $\varepsilon_{m}$ (mm/mm)
Twaron 2200	1	25.3	3.8	96.14	552.6	42,777	0.0129
	2	25.3	4	101.2	538.2	42,013	0.0128
	3	25.3	3.95	99.36	526.9	41,366	0.0127
				Average	539.2	42,052	0.0128
Kevlar 49	1	25.4	3.75	95.25	451.5	44,602	0.0101
	2	25.3	3.75	94.86	485.3	44,647	0.0109
	3	25.35	3.8	96.33	448.3	45,556	0.0098
				Average	461.7	44,935	0.0103
Pyrofil TR30 S	1	25.1	2.3	57.73	747.6	75,866	0.0099
	2	25.15	2.3	57.85	746.7	77,810	0.0096
	3	25.1	2.3	57.73	709.2	81,605	0.0087
				Average	734.5	78,427	0.0094
HR 12K	1	24.7	5.25	129.68	692.7	96,049	-
	2	25.05	5.05	126.5	680.3	85,343	0.0080
	3	24.75	5.3	131.18	684.9	89,136	-
				Average	680.3	90,176	0.0080
Dyneema SK 60	1	25	3.45	86.25	383.7	10,640	0.0361
	2	25	3.45	86.25	367.3	12,358	0.0297
	3	25.05	3.45	86.42	332.9	12,587	0.0264
				Average	361.3	11,862	0.0307

## Appendix A

The dimensions (in mm) of the strikers used in the conducted research are shown in Figure A1.
